# Soluble CEACAM8 Interacts with CEACAM1 Inhibiting TLR2-Triggered Immune Responses

**DOI:** 10.1371/journal.pone.0094106

**Published:** 2014-04-17

**Authors:** Bernhard B. Singer, Lena Opp, Annina Heinrich, Frauke Schreiber, Ramona Binding-Liermann, Luis Carlos Berrocal-Almanza, Kerstin A. Heyl, Mario M. Müller, Andreas Weimann, Janine Zweigner, Hortense Slevogt

**Affiliations:** 1 Institute of Anatomy, University Hospital, University Duisburg-Essen, Essen, Germany; 2 Institute of Microbiology and Hygiene, Charité - Universitätsmedizin Berlin, Campus Benjamin Franklin, Berlin, Germany; 3 ZIK Septomics, University Hospital Jena, Jena, Germany; 4 Labor Berlin - Charité Vivantes Services GmbH, Berlin, Germany; 5 Institute of Laboratory Medicine, Clinical Chemistry and Pathobiochemistry of the Charité Universitätsmedizin Berlin, Berlin, Germany; 6 Institute of Hygiene and Environmental Medicine, Charité - Universitätsmedizin Berlin, Campus Benjamin Franklin, Berlin, Germany; The University of Texas Medical School at Houston, United States of America

## Abstract

Lower respiratory tract bacterial infections are characterized by neutrophilic inflammation in the airways. The carcinoembryonic antigen-related cell adhesion molecule (CEACAM) 8 is expressed in and released by human granulocytes. Our study demonstrates that human granulocytes release CEACAM8 in response to bacterial DNA in a TLR9-dependent manner. Individuals with a high percentage of bronchial lavage fluid (BALF) granulocytes were more likely to have detectable levels of released CEACAM8 in the BALF than those with a normal granulocyte count. Soluble, recombinant CEACAM8-Fc binds to CEACAM1 expressed on human airway epithelium. Application of CEACAM8-Fc to CEACAM1-positive human pulmonary epithelial cells resulted in reduced TLR2-dependent inflammatory responses. These inhibitory effects were accompanied by tyrosine phosphorylation of the immunoreceptor tyrosine-based inhibitory motif (ITIM) of CEACAM1 and by recruitment of the phosphatase SHP-1, which could negatively regulate Toll-like receptor 2-dependent activation of the phosphatidylinositol 3-OH kinase-Akt kinase pathway. Our results suggest a new mechanism by which granulocytes reduce pro-inflammatory immune responses in human airways via secretion of CEACAM8 in neutrophil-driven bacterial infections.

## Introduction

The recruitment of neutrophils is one of the most important components of the initial, innate immune response of the human lung to bacterial infections [1]. The airway epithelium serves as the first line of respiratory mucosal defense. Toll-*like* receptor (TLR) 2, expressed on the apical surface of airway epithelial cells, is particularly important for the detection of inhaled bacteria in the human airways and the initiation of the innate immune response [2]. Neutrophils also express all TLRs except TLR3 [3]. Despite their active role in the pro-inflammatory immune response, neutrophils are part of the cellular network that orchestrates the resolution of inflammation by secreting a variety of molecules that possess anti-inflammatory effects in order to avoid tissue damage [3]. However, the crosstalk seen in the course of bacterial infection between neutrophil granulocytes and the airway epithelium for relieving inflammation, as well as decreasing their recruitment, are not well understood.

The carcino-embryonic antigen-related cell adhesion molecule (CEACAM)8, often better known as CD66b, encodes a glycosylphosphatidylinositol (GPI)-linked glycoprotein, which is exclusively expressed by human granulocytes [4]–[6]. CEACAM8 belongs to the carcinoembryonic antigen (CEA) family of the immunoglobulin superfamily. CEACAMs are involved in various intercellular-adhesion and cellular signaling-mediated effects modulating immune responses which are associated with the binding of pathogens, inflammation as well as growth and/or differentiation of normal and cancerous cells [7]. CEACAM8 is stored in specific vesicles of granulocytes and acts as a marker for specific vesicles for exocytosis [8]. Secretion has been shown to be induced by Phorbol-12-myristate-13-acetate (PMA) [9], [10]. Interestingly, no homolog for CEACAM8 has been identified in rodents, suggesting that there may be a strong selection pressure (e.g., exposure to microorganisms or parasites) during the evolution of molecules of the CEA family [11]. The soluble form of CEACAM8 binds to CEACAM1, a trans-membrane-bound molecule expressed by certain normal epithelial, endothelial, different leukocyte subpopulations and some cancer cells [12]. CEACAM1 bears an immunoreceptor tyrosine-based inhibitory motif (ITIM) in its intracellular domain known to be important for the initiation of the CEACAM1 signaling [7]. We recently demonstrated that CEACAM1 co-localizes with TLR2 on the surface of bronchial epithelium. Engagement of CEACAM1 by the *Moraxella catarrhalis* surface protein UspA1 dampened the TLR2-induced immune response initially triggered by the pathogen. Our data suggested that the interaction of *M. catarrhalis* with CEACAM1 might serve as immune evasion mechanism for this and other CEACAM1 binding pathogens which may contribute to their colonization of the airways of the lower respiratory tract. [13]–[15]. In pulmonary epithelial cells the CEACAM1-dependent co-inhibitory function of TLR2 was mediated by tyrosine phosphorylation of the ITIM and then by recruitment of the phosphatase SHP-1, which, in turn, all negatively regulated TLR2-dependent activation of the phosphatidylinositol 3-OH kinase-Akt kinase pathway. Consecutively, we hypothesized that CEACAM8 released by activated granulocytes might also diminish the TLR2-dependent immune response by interacting with the CEACAM1 of the pulmonary epithelium, favoring the resolution of inflammation. In the study reported here, we demonstrate that soluble CEACAM8 is released by human granulocytes in response to bacterial DNA. Soluble recombinant CEACAM8-Fc induces negative regulatory signals by interacting with CEACAM1, which is expressed on human pulmonary epithelium to inhibit TLR2 receptor signaling of the human airways.

## Materials and Methods

### Cells

Normal human bronchial epithelial cells (NHBEs) were obtained from LONZA (Lonza Group Ltd, Switzerland). NHBEs were plated in bronchial epithelial cell basal medium supplemented with recommended supplements (BEBM and BEGM, Lonza). Cells were grown to 80% confluence in pre-coated, 75-cm^2^ flasks (BD Bioscience) and cultured in pre-coated, 6- and 48-well culture plates (BD Biosciences) until confluence (100%) as described in the manufacturer's protocol. All experiments were performed with cells passage 2–8. A549 epithelial cells (type II alveolar cells) were obtained from DSMZ (DSMZ GmbH, Braunschweig, Germany) and cultured in DMEM (Gibco) supplemented with 10% fetal calf serum (FCS) (Gibco) and 1% Glutamine (PAA Laboratories GmbH, Pasching, Austria). Cells were grown to 80% confluence in 75-cm^2^ flasks (BD Falcon) and then cultured in six 48-well plates (BD Falcon) and 25-cm^2^ flasks (TPP, Trasadingen, Switzerland) until three-six days after confluence [6]. A549 cells were stably transfected either with pcDNA3.1 (A549-vec) or pcDNA3.1-CEACAM1-4L plasmids (A549-human-CEACAM1-4L) utilizing the Lipofectamin 2000 approach (Invitrogen) according to the manufacturers protocol. Following G418 selection (Gibco), transfected cells were sub-cloned and tested for the presence of CEACAM1 by flow cytometry. Neutrophils were isolated from the peripheral blood of healthy donors after a written informed consent document was signed. This procedure was approved by the ethic committee of the Medical Faculty of the Charité-Universitätsmedizin Berlin. After sedimentation of the erythrocytes by using dextran, neutrophils were isolated by Ficoll density gradients. After lysis of the remaining erythrocytes, neutrophils were harvested, washed with PBS and re-suspended in DMEM (Gibco, Pascagoula, MS) and 10% FCS at a cell concentration of 6×10^6^ in 500 µl, and pre-incubated with 50 ng/ml GM-CSF (ImmunoTools, Friesoythe; Germany) at 37°C and 5% CO_2_ for 90 min. After pre-incubation the inhibitory reagents (10 µM Pan Caspase fmk Inhibitor Z-VAD, R&D; 1 µg/ml Cycloheximide (CHX), Sigma; 0,5 µg/ml Cytochalasin D (CD), Calbiochem and 10 µM Pan-MMP Inhibitor GM-6001, Calbiochem) were added for 1 h, and the cells were then stimulated with 20 ng/ml Phorbol Myristate Acetate (PMA, Sigma) for 1 h. Cells that were only pre-incubated with CD were stimulated with CpG-ODN instead of PMA for 14 h. Granulocytes without inhibitory reagents were incubated as described previously [16] with the following reagents for 14 h at 37°C and 5% CO_2_: 20 ng/ml PMA (Sigma), 5 ng/ml tumor necrosis factor α (TNFa, R&D Systems), 10 µg/ml Pam_3_Cys (P3C, Enzo Life Sciences), 100 ng/ml Poly (I∶C) (P∶IC, Imgenex), 1 µg/ml Flagellin (Fl), 10 µg/ml Resiquimod-848 (R848, Enzo Life Sciences), and 100 µg/ml unmethylated CpG-ODN (Invivogen). The CEACAM8 release in the supernatants of the stimulated granulocytes was measured by CEACAM8 monospecific ELISA.

### Bronchio-alveolar fluid

BALF samples were obtained from 165 individuals in whom bronchoscopy was performed for different diagnostic or therapeutic purposes. All individuals underwent bronchoscopy following standard diagnostic procedures in the Department of Infectious Diseases and Pulmonary Medicine of the Charité-Universitätsmedizin Berlin. The patients received written study information by the treating physician and signed a written informed consent about the performance of a diagnostic bronchio-alveolar lavage and the subsequent analysis of the collected samples. The written informed consent remained in the patient's file. The investigators received residual material of the samples without any personal information or clinical data of the patients. The study documents and this procedure was proven and accepted by the ethic committee of the Medical Faculty of the Charité-Universitätsmedizin Berlin. BALF was centrifuged at 300 *g* for 10 min, and supernatants were then collected and stored in aliquots at −80°C until processing for ELISA. Cells were suspended in PBS and counted using a hemochromocytometer chamber. Differential cell counts were obtained through cytocentrifugation (Cytospin, Shandon Southern Instruments) of a small aliquot of BAL cell suspension. The cells were then air-dried and stained, followed by manual differential counting of cells after staining with May-Grunwald and Giemsa solution. At least 300 nucleated white blood cells were counted in each cytocentrifugation preparation [17]. The neutrophil count in the BAL was categorized as follows: normal 0.5–3.9%, moderately elevated 4.0%–14.9% and high ≥15.0% [17], [18].

### Production of soluble CEACAM8-Fc and rat-CEACAM1-Fc

The cDNA, which encodes the extracellular domain of CEACAM8 (N-, A1- and B1 domain) or rat CEACAM1 fused to human heavy chain Fc domain (CEACAM8-Fc, ratCEACAM1-Fc), was cloned into the pcDNA3.1(+) expression vector (Invitrogen, San Diego, CA), sequenced and stably transfected into HEK293 cells. The Fc chimeric proteins were accumulated in serum-free Pro293s-CDM medium (Lonza) and were recovered by Protein A/G-Sepharose affinity Chromatography (Pierce). Both proteins were then blotted by SDS-PAGE and stained by Coomassie blue demonstrating an equal amount and integrity of the used fusion proteins (data not shown). Endotoxin levels of the different Fc-construct preparations were determined using the Pyrogent plus Gel-Clot assay (Lonza) and were found to be below the detection limit of 0.06 U/ml.

### CEACAM8 and CEACAM6 Sandwich-ELISA

After stimulation, granulocytes were centrifuged at 200 *g* and room temperature for 10 min. The supernatants were again centrifuged for 35 min and at 16.000 *g* at 4°C. Secreted CEACAM8 and CEACAM6 were assessed in the supernatants. 96-well micro titer plates (MaxiSorb TM plates, Nunc, Denmark) were coated for 2 h at room temperature with 5 µg/ml rabbit-anti-CEA-antibody (Dako, Biomol GmbH, Hamburg, Germany) diluted in PBS. After washing the plate twice with 0.05% Tween (Carl Roth GmbH und Co KG, Karlsruhe, Germany) in PBS, all unbound sites were blocked for 2 h at room temperature with PBS containing 3% bovine serum albumine (Carl Roth GmbH). To quantify the CEACAM8 (B.B. Singer) in the supernatants, a standard curve was prepared by making serial dilutions of rhCEACAM8-Fc (from 0 ng/ml to 100 ng/ml) and rhCEACAM6-Fc (from 0 ng/ml to 100 ng/ml). The standard and the undiluted samples were incubated over night at 4°C. After washing three times, 10 µg/ml of the detection antibody mono-specific for CEACAM8 (mAb 6/40c, B.B. Singer) and mono-specific for CEACAM6 (1H7-4B, B.B. Singer) were added. Thereafter, plates were washed three times and supplemented with secondary horse radish peroxidase (HRP)-coupled goat anti-mouse antibodies (Dianova, Hamburg, Germany) for 2 h followed by three washing steps. The last washing step was performed with PBS only. Then 100 µl TMB-X-tra substrate (Biotrend Chemikalien GmbH, Cologne, Germany) was added and incubated for approximately 15 min. Incubation was stopped by 20 µl of 2 N H_2_SO_4_ (Carl Roth), and the optical density was read at 450 nm in a micro-plate reader (Tecan). All antibodies and the standard curve were diluted in PBS containing 1.5% BSA. The minimal detectable dose for the results of this CEACAM8 and CEACAM6 Sandwich-ELISA was calculated by adding two standard deviations to the mean optical density value of 20 zero standard replicates and determining the corresponding concentration from the standard curve. The minimal detectable dose for these CEACAM8 and CEACAM6 tests generated by this method was 0.84 ng/ml and 0.68 ng/ml, respectively. Cross-reaction with other human, rat and mouse CEACAM proteins was ruled out.

### IL-8 and IL-6 ELISA

IL-8 secreted by NHBE cells: A549 cells; and granulocytes and IL-6 secreted by NHBE cells were evaluated using a commercially available sandwich-ELISA kit according to the manufacturer's protocol (BD Biosciences).

### Flow cytometry

Human granulocytes, 0.5×10^6^ cells, were incubated with 4 µg of the primary antibody anti-human CD62L (L-Selectin, BD Pharmingen) diluted in DMEM (Gibco) and 10% FCS (Gibco) for 1 h at 4°C. Subsequently, samples were washed twice with DMEM containing 3% FCS and incubated with FITC-coupled anti-mouse F(ab)2 (Invitrogen, dilution 1/50) for 30 min at 4°C in the dark. Background fluorescence was determined using isotype-matched immunoglobulins, instead of specific primary antibodies.

To analyse the surface expression of TLR2 and CEACAM1 on NHBE and A549, cells were first fixed for 20 min in 2% formaldehyde on ice. After blocking with 10% FCS in PBS for 10 min, 0.5×10^6^ cells were incubated with 10 µg/ml mouse anti-TLR2 monoclonal antibody (mAb) (Acris Antibodies GmbH, Herford, Germany) or 3.5 µg/ml anti-CEACAM1 mAb 18/20 (B.B. Singer, Essen, Germany) for 45 min in PBS containing 10% FCS at room temperature. Afterwards, samples were washed twice with PBS containing 10% FCS and incubated for 30 min in the dark with PE-coupled goat anti-mouse F(ab)_2_ (dilution 1/50) at room temperature. After two washing steps in PBS and 0,2% Tween and one washing in PBS, the samples were measured in a FACSCalibur instrument (BD Biosciences, San Diego, CA) and the data were analysed using the CellQuest Pro software (BD Biosciences). Background fluorescence was determined using isotype-matched immunoglobulins instead of specific primary antibodies.

For the quantification of apoptosis, cells were harvested and stained for the appearance of phosphatidylserine exposed on the extracellular side of the plasma membrane. In brief, cells were treated with fluorescein-conjugated annexin V and propidium iodide according to the manufacturer's instructions on the Annexin V-Kit (Miltenyi Biotec, Bergisch Gladbach, Germany).

### Cell-based ELISA

First, 100 µL cell culture media with 15,000 A549-vec and A549-CEACAM1-4L cells, respectively, were seeded into each well of a 96-well cell culture micro-plate. Cells were incubated overnight in a cell culture incubator at 37°C to gain sparse cell monolayers [6]. Then, cell culture media was removed by flipping the plate up-side-down and 100 µl CEACAM8-Fc with indicated concentration diluted in cell culture media was added and kept on ice for 2 h. Rat CEACAM1-Fc (10 µg/ml) served as a negative control. In addition, we verified the presence or absence of CEACAM1 in A549-vec and A549-CEACAM1-4L cells utilizing 10 µg/ml of the anti-human CEACAM1 detecting mAb B3-17 and isotype matched IgG as negative control. Then, micro-plates were washed three times with cell culture media and samples were incubated with 100 µl HRP-coupled goat anti human-Fc pAb (1∶10,000) or HRP-coupled goat anti mouse antibody (Jackson Immunoresearch) diluted in cell culture media for 1 h on ice. Finally, the plates were washed three times and developed under protection against light using 100 µl TMB (Sigma) for approximately 20 min at room temperature. The reaction was stopped by 100 µl 0.2 M H_2_SO_4_ and the absorbance was detected at 450 nm in a Sunrise-ELISA reader (Tecan, Crailsheim, Germany). All measurements were performed in triplicates.

### Chromatin Immunoprecipitation (ChIP)

ChIP assays were performed as previously described [19]. A549 cells were stimulated at 37°C and 5% CO_2_ with Pam_3_Cys alone or in combination with soluble CEACAM8-Fc. After 1 h, the culture medium was removed, 1% formaldehyde (Roth) was added to fix the cells. Cells were incubated in ice-cold 0.125 M glycine (Roth) in PBS, washed twice in PBS, and then rapidly collected in ice-cold PBS. The cells were lysed in ChIP RIPA buffer (10 mM Tris-HCL, pH 7.5, 150 mM NaCl, 1% Nonidet P-40, 1% desoxycholic acid, 0.1% SDS and proteinase inhibitory cocktail set I), and the chromatin was sheared by sonification. The lysates were cleared by centrifugation and immuno-precipitations from soluble chromatin, which was carried out overnight at 4°C in the end-to-end shaker. Antibodies were purchased from Santa Cruz Biotechnology (200 µg/ml anti p65 and anti polymerase II). Immune complexes were collected with protein A/G-agarose for 60–120 min and washed twice with ChIP-RIPA buffer, once with a high salt buffer (2M NaCl, 10 mM Tris aminomethane, pH 7.5, 1% Nonidet P-40, 0.5% desoxycholic acid, 1 mM EDTA), then again with a ChIP RIPA buffer, and finally with a TE-buffer (10 mM Tris aminomethane, 1 mM EDTA). Immune complexes were extracted in elution buffer (1 TE buffer containing 1% SDS) by shaking the lysates for 15 min at 1200 rpm, 30°C. They were then digested with 0,5% RNase and diluted in TE-buffer for 30 min at 37°C and 5% CO_2_ (also input after adding 1% SDS). After proteinase K (2.5%) digestion (12 h at 37°C and 6 h at 65°C), all samples were stored at 4°C. On the next day, DNA was extracted using a PCR purification kit (Quiagen, Hilden, Germany). IL8 promoter DNA was amplified by PCR using Perfect Taq plus (5Prime) polymerase. The PCR conditions were 95°C for 5 min and 30 s for denaturation, annealing for 30 s at a variable temperature, elongation at 72°C for 1 min and 10 min. PCR products were separated by agarose gel electrophoresis and detected by ethidium bromide staining of gels. Equal amounts of input DNA was controlled by gel electrophoresis. The following promoter-specific primers for IL8 were used: sense, 5′-AAG AAA ACT TTC GTC ATACTC CG-3′; antisense, 5′-TGG CTT TTT ATA TCA TCA CCC TAG-3′

### Co-Immunoprecipitation (Co-IP)

For determination of CEACAM1 tyrosine phosphorylation and its putative association with SHP1, 5×10^6^ A549 cells were incubated for 5 min at 37°C and 5% CO_2_ in the presence of DMEM medium, DMEM medium containing 100 ng/ml of the TLR2 agonist Pam_3_Cys (P-3-C, Enzo Life sciences) in or not in combination with 100 ng/ml rh soluble CEACAM8-Fc (B.B. Singer) and 4 mM pervanadate (Sigma) as the previously described positive control [15]. As a negative control, rat CEACAM1 (ratCEACAM1-Fc) was used. In addition the potential of the antibody 18/20 to phosphorylate CEACAM1 compared to soluble CEACAM8-Fc was studied. An IgG antibody was used as control. Cells were then lysed in ice-cold lysis buffer containing 50 mM Tris-HCl (pH 7.5) (Roth), 1% Nonidet p-40 (Fluka Biochemica), 0.1% SDS (Roth), 5 mM sodium phosphate (Roth), 1 mM EDTA (Roth), 1 mM EGTA (Roth), 50 mM sodium fluoride (Serva), proteinase inhibitor cocktail set I (Calbiochem) and phosSTOP (Roche). Lysates were sonicated and centrifuged for 30 min at 4°C and 18 620 *g*. Supernatant were incubated with 5 µg/ml of primary polyclonal rabbit anti-CEACAM1 antibody (mAB 18/20), as described previously [15], for 1 h at 4°C together with Protein-A/G-Agarose over night at 4°C in the end-to-end shaker. The beads were precipitated by centrifugation (20 sec, 300 *g*) and washed four times with RIPA buffer. Subsequently, the immuno-precipitates were further analyzed by Tricine-PAGE and immuno-blotting. Tyrosine phosphorylation of CEACAM1, co-precipitation of SHP1 and precipitated CEACAM1 were detected by the phospho-tyrosine-specific mAb 4G10 (Millipore, Massachusetts, USA), anti-SHP1 (clone 1SH01, Calbiochem) and mAb 18/20, respectively. Immunoblots were incubated with the HRP-coupled secondary goat anti-mouse Ig antibody (Jackson Immuno Research), developed by ECL (AppliChem GmbH, Darmstadt, Germany), documented with Fuji LAS3000 imaging system and analyzed utilizing Fuji Image Gauge 3.45 software.

### Immunoblot

A549 cells were infected as indicated, lysed and sonicated in buffer containing Tris-HCL (Carl Roth), 1% Nonidet (Fluka Biochemica), inhibitory cocktail (Calbiochem) and phosSTOP (Roche). The protein concentration was determined via Bradford (Serva) and equal amounts of the samples were subjected to SDS-PAGE and blotted on Hybond-ECL membrane (Amersham Pharmacia Biotech Europe GmbH, Nümbrecht, Germany). Immuno-detection of proteins was carried out with the primary antibody phospho-akt Ser 473 (Cell signaling technology) diluted 1∶500, as well as the corresponding HRP-labeled secondary antibodies (Santa Cruz, California, USA). In all experiments, beta-actin (Santa Cruz), or β-tubulin (Abcam, Cambridge, UK) were measured after blot stripping on the same membrane to confirm equal protein load. After incubation with the HRP-coupled secondary antibody, the membrane was developed with enhanced chemiluminescence (ECL; Thermoscientific/Pierce, Rockford, USA). The reaction was monitored utilizing a Fuji LAS3000 imaging system (Fujifilm).

### RNA interference in A549 cells

A549 cells were transfected with siRNA targeting CEACAM1 (CEACAM1 smart pool, Thermo Scientific Dharmacon) and with a control non-silencing siRNA by using Lonza Nucleofector Kit V (Lonza, Cologne, Germany) according to the manufacturer's protocol (Nucleofector™ Solution V, Nucleofector™ program G-16) with 2 µg of siRNA in 100 µl solution V per 10^6^ cells. 96 h after siRNA transfection, cells were stimulated with Pam_3_Cys and rhCEACAM8-Fc as indicated overnight and supernatants were analysed for IL-8 by ELISA as described previously.

### Statistical analysis

**Figure 1:**
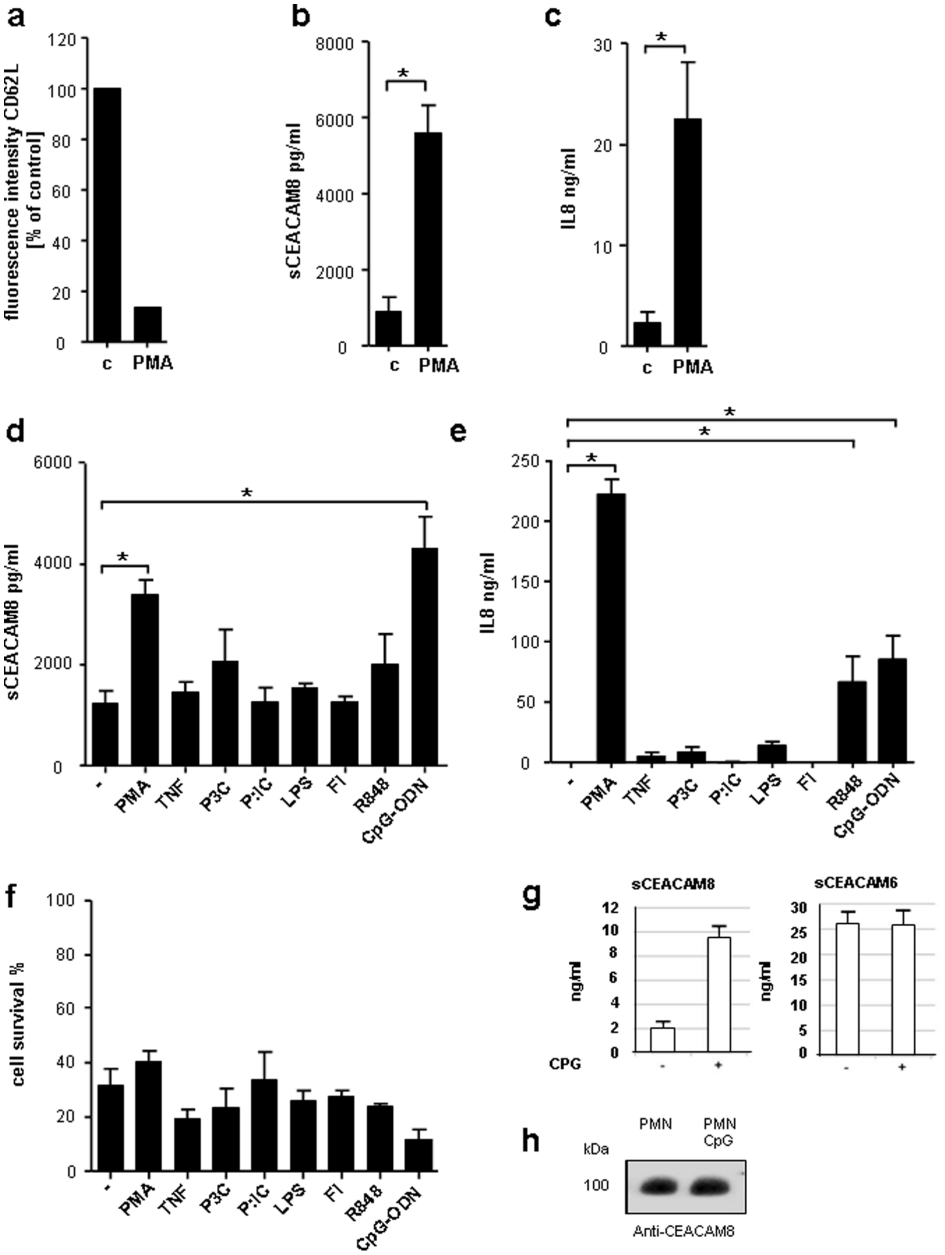
Analysis of CEACAM8 released by stimulated human granulocytes. (a) In all experiments granulocytes were pre-incubated using 50 ng/ml GM-CSF for 90 min. Expression of CD62L on human granulocytes was assessed by FACS analysis after treatment with 20 ng/ml PMA for 1 h. Shown is fluorescence intensity as percent of control. Secreted CEACAM8 (sCEACAM8) (b) and IL8 (c) in supernatants of granulocytes incubated for 1 h with 20 ng/ml PMA measured by ELISA. (d) ELISA for released CEACAM8 in supernatants of granulocytes treated for 14 h with 20 ng/ml PMA, 5 ng/ml tumor necrosis factor α (TNFα), 10 µg/ml Pam_3_Cys (P3C), 100 ng/ml Poly(I∶C) (P∶IC), 1 µg/ml Flagellin (Fl), 10 µg/ml Resiquimod-848 (R848) or 100 µg/ml non-methylated CpG (CpG-ODN). (e) IL8 secretion measured by ELISA from supernatants of granulocytes treated the same way as in d. (f) Cells were stimulated with agonists like in (d) and (e) for 14 h. Cell viability (Annexin V/Propidium iodide) was determined by FACS analysis (f). Bars represent the mean of viable cells as assessed by Annexin V/Propidium jodide staining. (g) Soluble CEACAM6 (sCEACAM6) and CEACAM8 (sCEACAM8) in supernatants collected from 10^7^ granulocytes treated for 14 hours with and without 100 µg/ml un-methylated CpG (CpG-ODN) were measured by ELISA. (h) To assess percentage of secreted CEACAM8 in relation to the total amount of cellular CEACAM8 lysates of CpG-ODN treated and untreated granulocyte were probed with the CEACAM8 specific mAb 80H3 by Western Blot. Data presented are mean ± s.e.m. of three different experiments performed in duplicates (b, c, d, e, f) or one of three identical experiments (a and h). * P<0.05.

**Figure 2:**
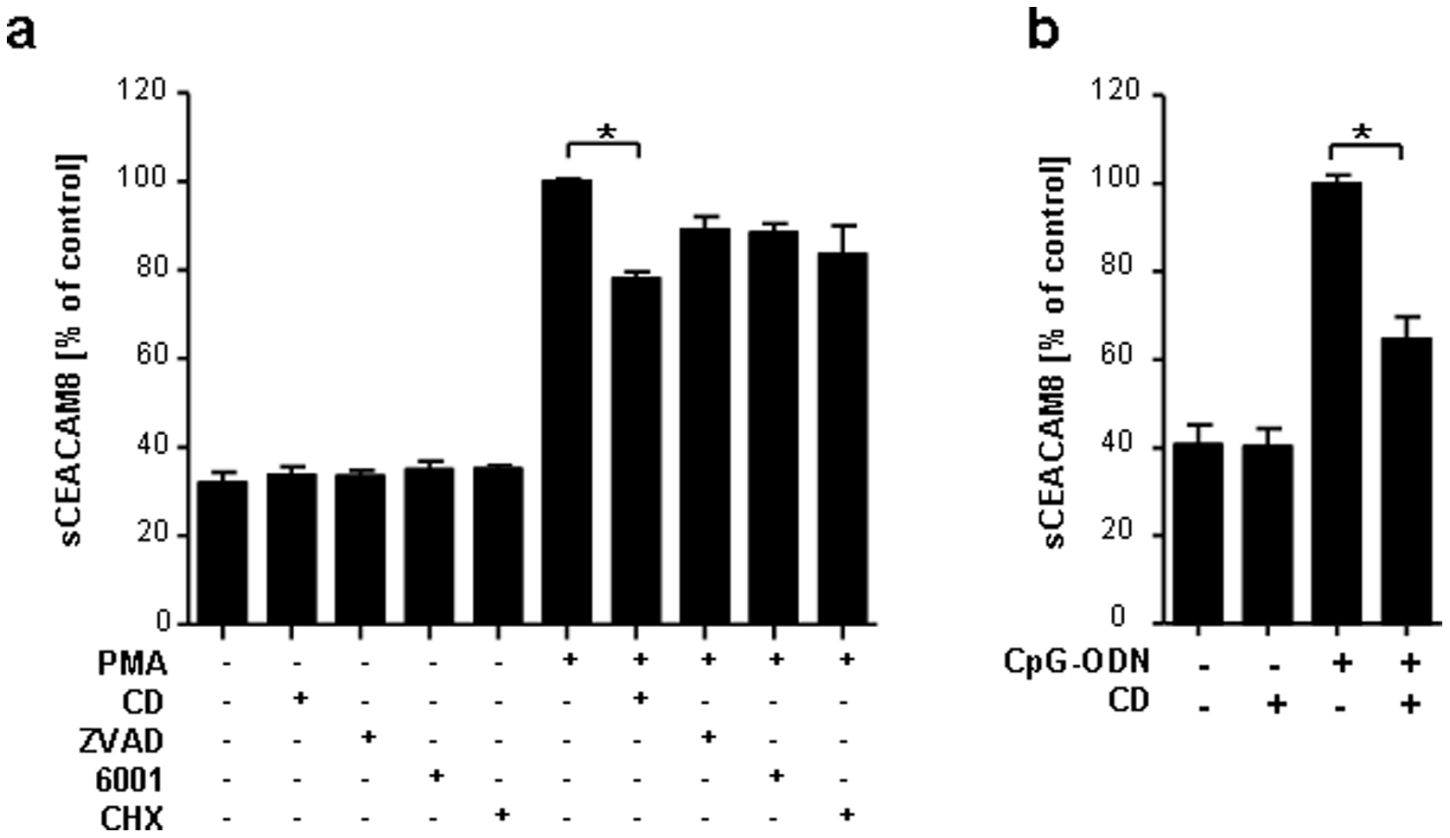
Secretion of sCEACAM8 is inhibited by the actin inhibitor cytochalasin D. (a) Released sCEACAM8 in supernatants of granulocytes measured by ELISA. Granulocytes were left untreated or pre-incubated for 1 h with one of the following stimuli: 0.5 µg/ml cytochalasin D (CD), 10 µM Pan caspase fmk Inhibitor Z-VAD (ZVAD), 10 µM Pan-MMP Inhibitor GM-6001 (6001) or 1 µg/ml cycloheximide (CHX) and then left untreated or incubated for 1 h with 20 ng/ml PMA. (b) CEACAM8 ELISA of supernatants harvested from granulocytes pre-treated with and without cytochalasin D (CD, 0.5 µg/ml) and 1 h stimulated with and without non-methylated CpG-ODN (b). Data presented are mean ± s.e.m. of three or four different experiments performed in duplicates. * P<0.05.

**Figure 3:**
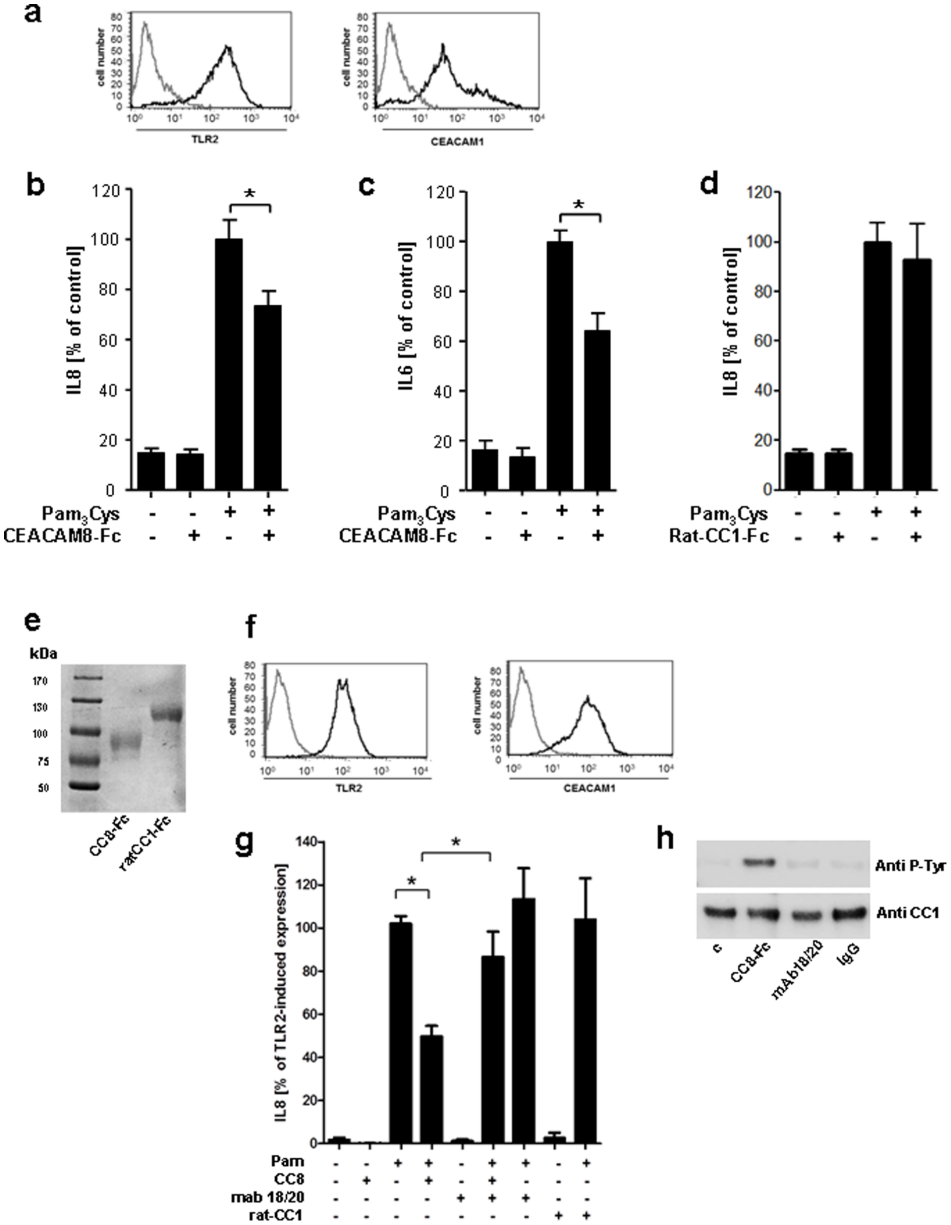
Soluble CEACAM8 interacts with membrane bound CEACAM1 and down-regulates the TLR2-triggered immune response on normal human bronchial epithelial (NHBE) cells. (a) FACS analysis of CEACAM1 and TLR2 (black lines) expression on the cell surface of NHBE cells. Grey lines represent isotype-matched control mAb staining. (b, c and d) ELISA for IL8 and IL6, respectively, in supernatants of NHBE cells incubated with Pam_3_Cys alone, with Pam_3_Cys plus soluble CEACAM8-Fc and rat-CEACAM1-Fc, respectively (100 ng/ml each) or left untreated for 16 h. (e) 50 ng purified protein of CEACAM8-Fc and ratCEACAM1-Fc were analyzed by SDS-PAGE and stained by Coomassie blue to demonstrate equal amount and integrity of the used fusion proteins. (f) FACS analysis CEACAM1 and TLR2 expression (black lines) on the surface of A549 cells. Grey lines represent the isotype-matched control. (g) IL-8 released into the supernatants of A549 cells pre-incubated for 1 h with anti-CEACAM1 (mAb 18/20) or left untreated followed by stimulation with Pam_3_Cys alone or with Pam_3_Cys and CEACAM8-Fc and ratCEACAM1-Fc, respectively (100 ng/ml) for 16 h. Samples were measured by commercial ELISA. IL8 secretion following treatment of the cells with Pam3Cys served as positive control. (h) A549 cells were challenged either with CEACAM8-Fc or with mAb 18/20 (20 ng/ml). The IgG antibody was used as control. Cell lysates were assessed after CEACAM1 immunoprecipitation with anti-phospho tyrosine mAb 4G10 (upper panel). Then membrane was stripped and re-probed with anti-CEACAM1 mAb (lower panel) to measure the amount of precipitated CEACAM1. Data presented in (a, e, f and h) are from one experiment representative of three independent experiments, and the data presented in (b, c, d and g) are mean ± s.e.m. of three different experiments performed in triplicates, * P<0.05.

**Figure 4:**
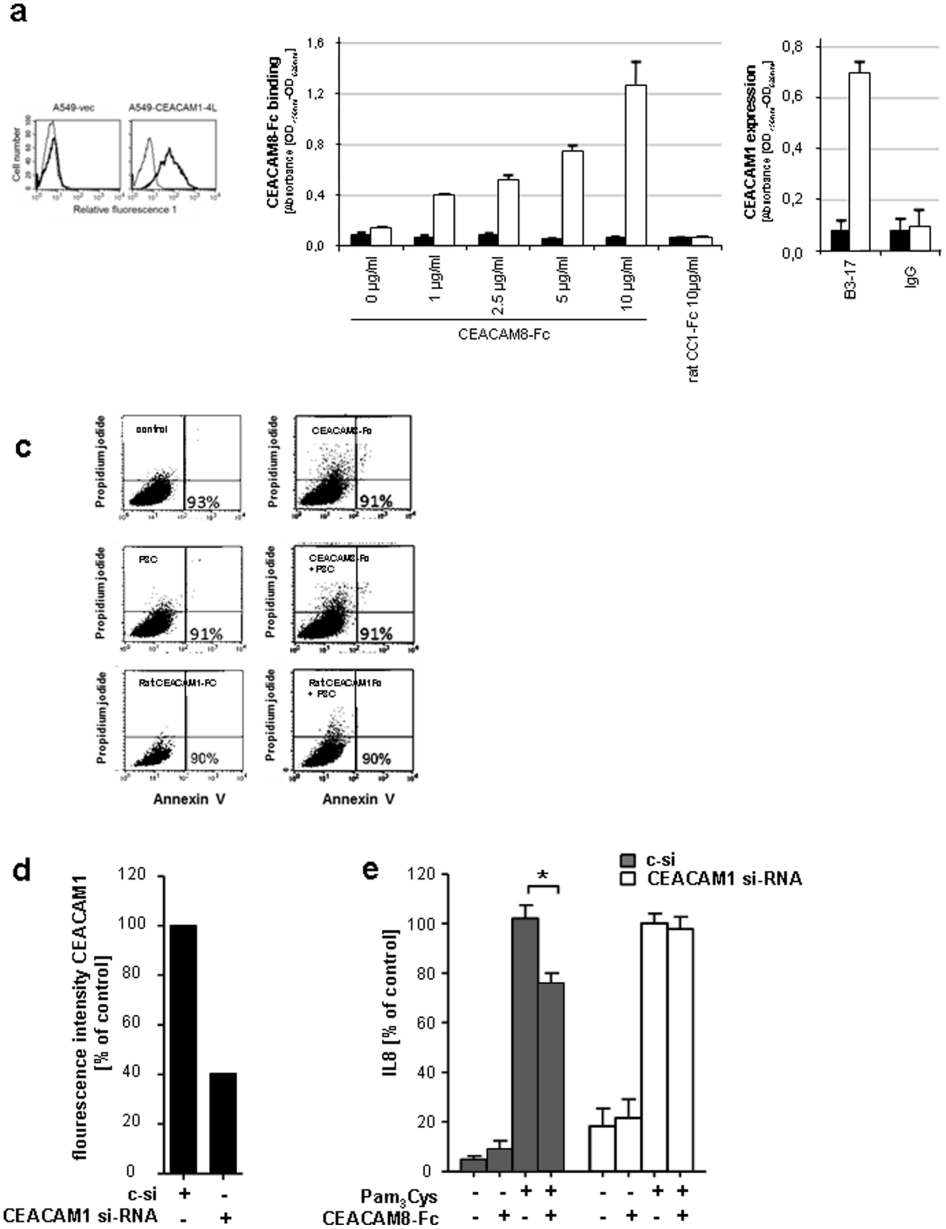
Interaction of soluble CEACAM8-Fc with CEACAM1 expressed on pulmonary epithelial cells mediates down-regulation of the TLR2-dependent immune response. (a) FACS analysis of CEACAM1 (bold lines) expression on the cell surface of sparse A549-CEACAM1-4L and vector-transfected A549 cells (A549-vec). Thin lines represent isotype-matched control mAb stainings. (b) Sparsely grown A549-CEACAM1-4L cells were challenged with recombinant CEACAM8-Fc as indicated (white bars, left panel). Sparsely grown A549-vec cells were used as a control (black bars, left panel). Rat CEACAM1-Fc neither binds to sparse A549-CEACAM1-4L nor A549-vec cells. The presence and absence of CEACAM1 on the cell surface of sparse A549-vec and A549-CEACAM1-4L was tested utilizing CEACAM1 specific mAb B3-17 (right panel). Isotype matched IgG served as negative control. Black bars = A549-vec, white bars = A549-CEACAM1-4L. (c) Viability of A549 cells 16 h after treatment with CEACAM8-Fc, Pam_3_Cys (P3C), or with Pam_3_Cys plus soluble CEACAM8-Fc and ratCEACAM1-Fc, respectively (100 ng/ml each) or left untreated, were stained with annexin V-FITC/propidium iodide and measured using flow cytometry. (d) A549 cells were transfected with CEACAM1-targeting siRNA or control siRNA (csi) for 96 h and expression of CEACAM1 was measured by FACS analysis. (e) IL-8 released by A549 cells transfected for 96 h with CEACAM1-specific or control siRNA and infected for 16 h with Pam_3_Cys alone or in combination with CEACAM8-Fc (100 ng/ml) were measured in the supernatants of the cells by ELISA (e). (a, c and d) Data are from one experiment representative of three independent experiments. (b, and e) Data presented are mean ± s.e.m. of three different experiments performed in duplicates. *P<0.05.

Data are shown as means ± S.E.M. for at least three independent experiments. A one-way ANOVA with Newman-Keul's post hoc test was used for comparison (Fig. 1b–g, Fig. 2a and b, Fig. 3b, c, d and g, Fig. 4b, e). The data in table 2 were analyzed with single variable logistic regression analysis to assess the relationship between predictors and outcome variable by obtaining the Odds Ratios (OR). A multi-variable logistic regression analysis was used to adjust for the effect of covariates on the outcome. All tests were two sided, and a p value<0.05 was considered statistically significant.

**Table 2:** Binary logistic regression analysis.

Predictor variables	β	Exp β (Odds ratio)	P value	95% C.I or OR
high vs normal	2,305	10,022	<0,0001	3,914–25,661
high vs moderate	1,84	6,3	0,0002	2,0–20,5
high vs normal/moderate	1,52	5,4	0,0001	2,0–15,7
moderate/high vs normal	1,301	3,675	<0,0001	1,897–7,117
moderate vs normal	0,456	1,578	0,241	0,736–3,385

The presence of detectable, secreted CEACAM8 in BALF was categorized as positive or negative and used as the outcome variable and the different categories of granulocyte count in the human BALF: normal, moderately elevated and highly elevated were used as predictor variables. The probability of having detectable secreted CEACAM8 in BALF related to one category or a combination of categories of the granulocyte count was then evaluated.

## Results

### CEACAM8 release by human granulocytes is partly induced by CpG-ODN

To assess the functional effect of PMA on human granulocytes 6×10^6^ freshly isolated PMNs were stimulated with PMA (20 ng/ml) for 1 h. Prior to all experiments, granulocytes were primed with 50 ng/ml GM-CSF for 90 min as described previously [16]. GM-CSF-related changes in CD62L expression and interleukin-8 (IL-8) cytokine release were ruled out by FACS analysis and ELISA, respectively (data not shown). Stimulation with PMA led to granulocyte activation, which was reflected by a considerable decrease in L-selectin (CD62L) cell surface expression (**Fig. 1a**). Subsequently, analysis on the secretion of CEACAM8 in the supernatant of PMA-stimulated human granulocytes was conducted. A strong release of CEACAM8, measured by ELISA, was found in the supernatant of the cells (**Fig. 1b**). An increase of IL-8 in the supernatants of the PMA-stimulated cells was also evident for concurrent granulocyte activation (**Fig. 1c**). In the next step, the role of different pattern recognition receptors for their regulatory function in the release of CEACAM8 receptor was investigated. Cells were stimulated with agonists for TLR2 (Pam_3_Cys), TLR3 (Poly∶IC), TLR4 (LPS), TLR5 (Flagellin), TLR7/8 (Resiquimod-848, (R848)), TLR9 (unmethylated CpG) and TNFα for 14 h. A significant difference in the level of released CEACAM8 was found in the supernatant of the cells. In response to TLR9-dependent cells stimulated with un-methylated CpG-ODN showed an increase release of CEACAM8 (**Fig. 1d**). Concurrent IL-8 secretion was, besides PMA stimulation, also highest after stimulation with CpG-ODN (**Fig. 1e**). Stimulation with TNFα, Pam_3_Cys and LPS also led to a slight, with R848 even a significant increase in IL-8 secretion, which was not associated with an increase in sCEACAM8 release. Cell viability (Annexin V/Propidium iodide) was determined by FACS analysis after each stimulation demonstrating that viability was reduced by approximately 60–90% of the cells (**Fig. 1f**). The differences in the amount of death granulocytes in the diverse treated samples seemed not to be related to the differences in the levels of secreted CEACAM8 (**Fig. 1f**). CEACAM6 is also expressed in human granulocytes and was described to interact with CEACAM1 [9]. To address the question whether CEACAM6 is also released by human granulocytes in a CpG-ODN-dependent manner we analyzed the supernatants of granulocytes treated and untreated with CpG-ODN for 14 h. As shown in figure 1g we confirmed the CpG-ODN-triggered secretion of soluble CEACAM8. We also found considerable amounts of CEACAM6 in the supernatants of the granulocytes. However, in contrast to the secretion of CEACAM8, CEACAM6 was not modulated by CpG-ODN-associated TLR9 activation (**Fig. 1g**). These data suggest that the mechanisms regulating the expression of soluble CEACAM6 are different compared to the CpG-ODN-associated regulation of soluble CEACAM8. It is well documented that CEACAM8 is stored in the neutrophilic vesicles and released in response to PMA [10], [20]. To assess the percentage of secreted CEACAM8 in relation to the total amount of cellular CEACAM8, cell lysates of CpG-ODN treated and untreated granulocyte were probed with the CEACAM8 specific mAb 80H3. We found no significant reduction in CEACAM8 protein levels in the Western Blot between non-stimulated and CpG-ODN stimulated samples (**Fig. 1h**). Zhao et al estimated the total cellular content of CEACAM8 in granulocytes to be 82.4+/−8.9 ng/10^6^ cells (mean +/− SE, n = 10), [20]. In our study we demonstrated that approximately 0.5–1 ng CEACAM8 was secreted by 1×10^6^ granulocytes. In accordance with this result, our data also suggest that the proportion of secreted CEACAM8 is too small to result in detectable differences quantitatively assessed using the Western Blot method.

### PMA and TLR9-dependent release of CEACAM8 is inhibited by Cytochalasin D

In a next step, the underlying mechanism of the CEACAM8 release in PMA-stimulated granulocytes pre-treated with or without cytochalasin D, the pan-caspase inhibitor Z-VAD fmk, the pan-metalloproteinase inhibitor GM-6001 and cycloheximide, respectively, were confirmed. The different inhibitors showed no significant difference in the release of CEACAM8 when compared with untreated cells, except for cytochalasin D, which is known to block the production and transport of secretory vesicles (**Fig. 2a**). Also the TLR9-triggered release of CEACAM8 was further investigated to establish whether it is also inhibited in the presence of Cytochalasin D. The CpG-ODN-induced release of CEACAM8 was considerably reduced by cytochalasin D (**Fig. 2b**).

### In human bronchial lavage fluid (BALF), individuals with a high granulocyte count are more likely to have detectable released CEACAM8

To evaluate a potential association between the presence of released CEACAM8 and the amount of granulocytes in human BALF of 165 patients in which a bronchoscopy was performed for different diagnostic or therapeutic purposes, the amount of granulocytes in BALF was measured using a hemochromocytometer chamber. Differential cell counts were obtained through cytocentrifugation (data not shown), and supernatants were assessed for the presence of released CEACAM8 by ELISA with a detection level for secreted CEACAM8 of 0.84 ng/ml. Results demonstrate that individuals with a high granulocyte count (≥15% of granulocytes) are significantly more likely to have detectable soluble CEACAM8 in the BALF than those with a normal (0–3.9%) or moderately elevated granulocyte count (14.9%), Odds Ratio (OR) 10 (95% CI 3,9–25,6) and OR 6,3 (95% CI 2,0–20,5) respectively (**Table 1** **and** **2**). This data show that the percentage of granulocytes in the BALF is a significant predictor for the release of CEACAM8 by activated granulocytes.

**Table 1:** Frequency counts of detectable secreted CEACAM8 and granulocytes in the human BALF.

Secreted CEACAM8 in human BALF	Granulocytes (% of nucleated cells in BALF)			
	Normal (0–3,9%)	Moderately elevated (4–14,9%)	Highly elevated (≤15%)	P value
CEACAM8 pos/neg (%)	31,3/68,7	41,9/58,1	82,1/17,9	<0,0001

The mean total number of granulocytes in the BALF was 4.7×10^6^/100 ml BALF (range 0–1.6×10^8^ granulocytes/100 ml BALF). The granulocytes were counted as the percentage of the total cells in the BALF and categorized as follow: normal 0.5%–3.9%, moderately elevated 4.0%–14.9% and highly elevated ≥15.0%. These percentages were compared between individuals with positive or negative secreted CEACAM8 in the BALF.

### Soluble CEACAM8-Fc treatment of primary normal human bronchial epithelial cells down regulates the TLR2-induced immune response

At confluence normal human bronchial epithelial (NHBE) cells showed considerable cell surface expression of CEACAM1 and TLR2 (**Fig. 3a**). The amount of IL-8 and IL-6 in the supernatants of NHBE cells stimulated with Pam_3_Cys alone or in a combination with recombinant human soluble CEACAM8-Fc were compared to evaluate the functional effect of the interaction between membrane bound CEACAM1 on NHBE and released CEACAM8 for the TLR2-dependent immune response. Stimulation of the cells with Pam_3_Cys in the presence of soluble CEACAM8-Fc resulted in a significant reduction in IL-8, as well as in IL-6 release when compared to stimulation of the cells with Pam_3_Cys alone (**Fig. 3b and c**). The N-domain of human CEACAM1 is composed of a single immunoglobulin variable (IgV)-like N-terminal (N) domain. In rats, different CEACAM1 alleles have been identified, which each differ considerably from humans. Therefore, rat CEACAM1 is known not to bind any pathogens or human CEACAMs and thus was used as control in the study presented here [21]. To rule out differences in the amounts of the used fusion proteins that might influence the results of our experiments, CEACAM8-Fc and rat-CEACAM1-Fc were analyzed by SDS-PAGE and stained by Coomassie blue. Both proteins demonstrated an equal amount (**Fig. 3e**). In a next step we demonstrated that the amount of IL8 secretion in the supernatants of NHBE cells either stimulated with Pam3cys alone or in the presence of rat CEACAM1-Fc showed no differences when measured by ELISA (**Fig. 3d**). These results suggest that soluble CEACAM8-Fc reduces the TLR2-dependent pro-inflammatory immune response in human pulmonary epithelium. To further investigate the regulatory mechanism of the sCEACAM8-CEACAM1 interaction in confluent pulmonary epithelial cells, RNA-mediated interference experiments were performed. The viability of NHBE cells was much lower during the gene knockout procedure because of the transfection reagents (data not shown). Therefore, the pulmonary epithelial cell line A549 was used, which has been used successfully before in experiments which efficiently accomplished target-gene silencing of CEACAM1 and TLR2 [14], [15]. First, the cell surface expression of CEACAM1 and TLR2 was confirmed (**Fig. 3f**). In a next step the release of IL-8 induced by Pam_3_Cys and its reduction in the presence of soluble CEACAM8-Fc were confirmed and were found to be similar in NHBE and A549 cells (**Fig. 3g**). In parallel treatment of the cells with Pam3cys in the presence of ratCEACAM1-Fc did not alter the TLR2-mediated immune response (**Fig. 3g**). The IL-8-dampening effect of soluble CEACAM8-Fc was confirmed by reversing the effect using incubation of the cells with the CEACAM1-blocking antibody 18/20 as has been previously described [15]. In addition, phosphorylation of epithelially expressed human CEACAM1 by the antibody 18/20 was ruled out by co-immuno-precipitation (**Fig. 3h**).

### Soluble CEACAM8-Fc binds to CEACAM1 expressed on A549 cells

To investigate the interaction of CEACAM1 with CEACAM8-Fc in a human pulmonary epithelial cell system we tested the binding of recombinant soluble CEACAM8-Fc to human CEACAM1 expressed on A549 cells. We compared sparsely grown CEACAM1 negative stably vector transfected A549 cells (A549-vec) with A549 transfectants that were stably transfected with CEACAM1-4L (A549-CEACAM1-4L). Cell surface expression of CEACAM1 was confirmed by flow cytometry (**Fig. 4a**). As illustrated by Figure 4b (left panel), we demonstrated a clear dose dependent binding of recombinant CEACAM8-Fc to membrane anchored CEACAM1 present in sparsely grown A549-CEACAM1-4L cells. In contrast, sparsely grown A549-vec cells that did not express CEACAM1 showed no binding of recombinant CEACAM8-Fc to these cells. Recombinant rat CEACAM1-Fc utilized as a control neither showed any binding to sparse A549-CEACAM1-4L nor to sparse A549-vec cells. Parallel to each cell-based ELISA, the presence or absence of CEACAM1 expression on A549-CEACAM1-4L and A549-vec was confirmed in both settings utilizing the CEACAM1 specific mAb B3-17 and isotype matched control Ig, respectively (Fig. 4b, right panel). Therefore our data corroborate the specific binding of CEACAM8 to membrane anchored CEACAM1 in human pulmonary epithelial cells and in principle are confirming the previous data of Jiang et al. [12].

In a next step, FACS analysis of annexin V-FITC and propidium iodide staining was performed to rule out that the reduction of the Pam_3_Cys triggered IL-8 secretion caused by soluble CEACAM8-Fc was related to changes in the cell viability. Neither CEACAM8-Fc nor the control rat CEACAM1-Fc with and without Pam_3_Cys did alter the survival rate if compared to untreated A549 cells (**Fig. 4c**). Furthermore, we confirmed that the CEACAM1-specific small interfering RNA (CEACAM1-si-RNA) constructs but not the non-silencing control siRNA (c-si) resulted in a significant reduction of cell surface expression of CEACAM1 on A549 cells, as measured by flow cytometry (**Fig. 4d**) and CEACAM1 mRNA in A549 cells, as determined by RT-PCR (data not shown). Using A549 cells transfected with control siRNA or CEACAM1-specific siRNA, the release of IL-8 triggered by Pam_3_Cys in the presence of soluble CEACAM8-Fc, but not that induced by Pam_3_Cys alone, was significantly increased by CEACAM1-specific siRNA (**Fig. 4e**).

### The inhibitory impact of the sCEACAM8 - CEACAM1 interaction on the TLR2-dependent immune response is mediated via tyrosine phosphorylation of CEACAM1 and the recruitment of SHP-1, which negatively regulated TLR2-dependent activation of the PI(3)K-Akt kinase pathway

**Figure 5:**
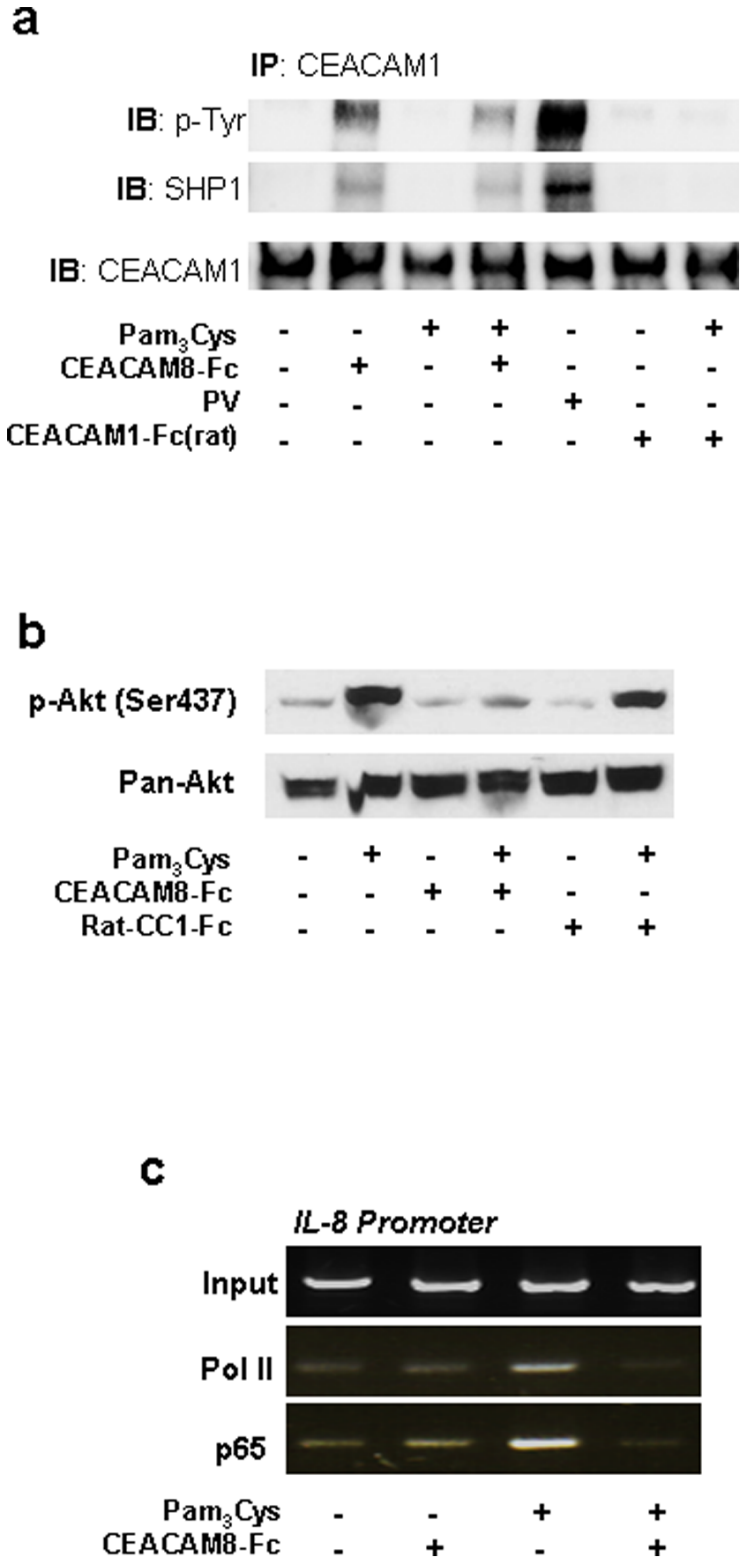
The inhibitory effect of the soluble CEACAM8 interaction with membrane bound CEACAM1 for the TLR2-dependent immune response resembles the UspA1-CEACAM1 interaction of *M. catarrhalis* in pulmonary epithelial cells. (a) Immunoprecipitation of CEACAM1 from A549 cell lysates. Cells were left untreated or were treated with Pam_3_Cys, Pam_3_Cys plus CEACAM8-Fc and ratCEACAM1-Fc, respectively (100 ng/ml). Stimulation with pervanadate served as positive control. Tyrosine phosphorylation of CEACAM1, co-precipitated SHP1 and total CEACAM1 were detected in this order by Western blot. (b) Immunoblot analysis of Akt phosphorylation in A549 cells treated with and without Pam_3_Cys alone or together with CEACAM8-Fc and ratCEACAM1-Fc, respectively (100 ng/ml each) for 60 min. Detection of Pan-Akt was used as loading control. (c) ChIP analysis of the binding of P65 and polymerase II (Pol II) to the IL8 promoter in A549 cells stimulated and unstimulated with Pam_3_Cys alone and together with CEACAM8-Fc (100 ng/ml). Data shown are from one experiment representative of three independent experiments.

To further explore signaling events triggered by the sCEACAM8-CEACAM1 interaction A549 cells were incubated with Pam_3_Cys alone or in the presence of soluble CEACAM8-Fc. Then cell were lysed and CEACAM1 was immuno-precipitated and analysed for its tyrosine phosphorylation status by immunoblot. Soluble CEACAM8-Fc alone or in the presence of Pam_3_Cys, but not Pam_3_Cys alone resulted in a CEACAM1 tyrosine phosphorylation (**Fig. 5a**). Soluble rat CEACAM1-Fc was used to rule out a stimulatory effect by putative Fc-receptors on the cells (**Fig. 5a**) [5]. Next, the recruitment of SHP-1 to phosphorylated CEACAM1 in response to stimulation of the cells with Pam_3_Cys in the presence or absence of soluble CEACAM8-Fc was investigated. SHP-1 immuno-precipitated together with phosphorylated CEACAM1 in those pulmonary epithelial cells incubated with soluble CEACAM8-Fc, but not in those incubated with Pam_3_Cys alone (**Fig. 5a**). Phosphorylation of the kinase Akt at the serine residue at position 473 (Ser473) is specifically dependent on PI(3)K activity and is reduced as a result of the inhibitory regulation of the UspA1-CEACAM1 interaction on the TLR2-dependent activation of the PI(3)K. In accordance with the hypothesis suggested earlier, immunoblot analysis showed considerable phosphorylation of Akt Ser473 in A549 cells stimulated with Pam_3_Cys alone, which decreased by pre-treatment with CEACAM8-Fc but not by ratCEACAM1-Fc (**Fig. 5b**). Next, by ChIP analysis a noticeable decrease of p65 and RNA polymerase II recruitment to the IL-8 promoter was seen in cells treated with Pam_3_Cys and soluble CEACAM8-Fc when compared to cells that were treated with Pam_3_Cys alone (**Fig. 5c**).

## Discussion

In the study presented here, for the first time we provide evidence that soluble CEACAM8 dampened the TLR2-triggered immune response of CEACAM1-expressing human pulmonary epithelial cells. The release of CEACAM8 by granulocytes was, at least partially, triggered by CpG-ODN in a TLR9-dependent manner. As confirmed in the study, CpG-ODN is known to induce IL-8 release and to trigger L-selectin (CD62L) shedding on the surface of human neutrophils [22]. In addition, CpG-ODN has also been demonstrated to enhance neutrophilic phagocyte activity [22]. However, bacterial DNA may persist in tissues, even in the absence of bacteria as in the human airways [22]–[24]. In our study we demonstrated that the release of CEACAM8 was induced by bacterial CpG-ODN treatment, which suggests a TLR9-dependent mechanism. These observations imply that for the resolution of bacteria-induced, pro-inflammatory immune responses after eradication of the pathogens, simply killing bacteria may not be enough in order to fully inhibit activation of neutrophils and surrounding tissues [22]. Shields et al recently suggested redefining a group of intracellular proteins as resolution-associated molecular patterns (RAMPs) which are released at times of cellular stress and which help to counterbalance the inflammatory effects of pathogen-associated (PAMPs) and damage-associated (DAMPs) molecular patterns [25]. According to this definition, it is likely that, during bacterial-induced airway infections, secretion of CEACAM8 by activated granulocytes in response to bacterial DNA may serve a resolution-associated mechanism to reduce further influx of neutrophils into the airways after sufficient killing of the pathogen. Our study further demonstrated that in human BALF the detection of released CEACAM8 is associated with a higher percentage of granulocytes. The incubation of pulmonary epithelial cells with CEACAM8-Fc induced a tyrosine phosphorylation of the ITIM of CEACAM1. This led to a recruitment of the phosphatase SHP-1, which negatively regulated TLR2-dependent activation of the phosphatidylinositol 3-OH kinase-Akt kinase pathway and subsequently cytokine and chemokine release by these cells.

The dampening of the TLR2-dependent immune response of human airway epithelium expressing CEACAM1 by soluble CEACAM8-Fc resembles the regulatory pathways recently identified for *M. catarrhalis* [15]. Mechanisms dampening inflammatory responses are interesting targets for subversion by microbes to reduce host sensing and avoiding recognition [8]. Therefore, Moraxella specific UspA1 expressed by the CEACAM1-binding pathogen *M. catarrhalis* resembles human soluble CEACAM8-Fc in its ability to reduce the TLR2-dependent immune responses of human airway epithelium. In addition to its role as a co-inhibitory receptor of TLR2 [15], CEACAM1 displays a co-inhibitory function in B and T cells [7], [26]. Further co-inhibitory functions of CEACAM1 for other receptors have been identified, such as G-CSFR mediated granulopoiesis [27]. A co-inhibitory function has also been demonstrated for CEACAM1 negatively regulating the TLR4-dependent IL-1β production in LPS activated neutrophils [28]. In contrast, two studies have suggested that ligation of CEACAM1 on B and T cells under cross-linking conditions in the presence of anti-BCR and anti-CD3, respectively, induces B and T cell activation [29], [30]. But the underlying mechanisms of activating effects of CEACAM1 on T cells have not been elucidated until now. Following the findings of our study, that soluble CEACAM8 functions as active ligand for CEACAM1, it becomes tempting to speculate that the interaction between secreted CEACAM8 with membrane-bound CEACAM1 might have an impact on far more receptors than TLR2 expressed on epithelial cells. However, more studies are needed in order to further clarify the role of sCEACAM8 released by granulocytes in the human airways and at other sites of infections.

In conclusion, we found that soluble CEACAM8 is released following CpG-ODN-dependent stimulation by human granulocytes. In addition our data suggest that soluble recombinant CEACAM8-Fc dampens the TLR2-triggered immune response by interacting with CEACAM1 expressing human airway epithelium. During bacterial airway infections, this interaction might serve as an important resolution-associated immune mechanism to further reduce neutrophil influx favoring the termination of inflammation and to avoid collateral tissue damage.
